# Health and Diet among People Living in an Isolated Area: Case Study of Pingelap Island in Pohnpei State, the Federated States of Micronesia

**DOI:** 10.3390/ijerph17217839

**Published:** 2020-10-26

**Authors:** Moeno Sakai, Minato Nakazawa, Delpihn Abraham

**Affiliations:** 1 Department of Public Health, Graduate School of Health Science, Kobe University, Kobe 654-0142, Japan; minato-nakazawa@umin.net; 2 Department of Health and Social Affairs, Pohnpei State Department of Health Services, Kolonia, Pohnpei FM96941, Micronesia; delabe08@gmail.com

**Keywords:** BMI, blood pressure, Pacific Island, diet, modernized

## Abstract

Pingelap Island in Pohnpei state is geographically isolated as the nearest island is 70 km away, and such geographical isolation is a challenge for public health due to the limited access to health services. This study aims to reveal the health situation on the island and investigate the influence of geographical isolation on health and diet. For that purpose, the result was compared with those who are living in a community on the main island of Pohnpei state (Mand) with the same ethnic background. Face-to-face interviews were conducted to collect data on demographics, diet, and behavior. Anthropometry and blood pressure measurements were also taken. A total of 98 (Pingelap = 50; Mand = 48) subjects participated in the study. The result showed that females, in particular, had a high prevalence of obesity (80.0% in Pingelap; 75.9% in Mand). However, no significant regional difference was found in both BMI and blood pressure, regardless of gender. Regarding diet, although the geographical location impacted food availability, the consumption of major imported foods did not show a significant regional difference. In conclusion, the geographical isolation did not significantly influence health and diet, but the majority of the study population displayed a high-risk burden of non-communicable diseases.

## 1. Introduction

Pingelap atoll is located 250 km away from Pohnpei island in the Eastern Carolines at 6°13′ N and 160°42′ W [1]. Pingelap atoll consists of three islets, namely, Pingelap Island (Pingelap), Deke Island, and Sukoru Island, which make up an area of 1.8 km^2^. However, out of the three, only Pingelap is inhabited. Pingelap is one of the islands of Pohnpei state, which consists of one main island (Pohnpei) and five small inhabited outer islands in the Federated States of Micronesia (FSM). A recent study indicated that the human settlement of Pingelap occurred in 1700–1550 BP at the latest [2], which is only a few centuries after the settlement of Pohnpei island.

The island has attracted attention due to its specific feature of achromatopsia, which is also referred to as color blindness. Especially, Morton et al. [3,4] conducted several studies on color blindness from the perspective of human genetics. The origin of colorblindness dates back to the 1770s with a population of approximately 1000. However, a typhoon named Lengkieki attacked the island, which resulted in the death of 90% of the population. Two weeks after the typhoon, the population on the island further decreased to approximately 30 due to a food shortage. Such shrinking followed by population growth caused a bottleneck effect in the island population, which was characterized by extremely unique genetic features. One of these features is complete color blindness (monochromacy), which remains in nearly 10% of the Pingelapese population. This prevalence is relatively high compared with the general population (1/30,000). The monochromacy of the Pingelapese can be traced back to a single male among the approximately 30 survivors [1,5,6]. In 1905, another typhoon hit the island. Although the typhoon did not claim lives, the tremendous destruction of vegetation produced near-starvation conditions. The German administration forced approximately 300 Pingelapese to move to Sokehs in Pohnpei island because of this damage and for political expediency after an uprising in Pohnpei. Independent of the forced movement, many Pingelapese migrated to Mand due to overcrowding in Pingelap in the 1950s [7,8]. In 1994, Oliver Sacks visited Pingelap island and Mand and published a book in 1998 about the visit entitled *The Island of the Colorblind*. The author stated that the Pingelapese tended to marry among themselves, even in Pohnpei. Therefore, genetic identity as Pingelapese seems to remain in Mand, which is partly supported by the fact that the prevalence of monochromacy was also high in Mand [5].

Not only the high prevalence of monochromacy but also the geographical isolation attracted researchers. The nearest island to Pingelap is 70 km away. Access to Pingelap remains very limited at present. A small aircraft (maximum passenger = eight) flies to the island from Pohnpei. However, the flight schedule is irregular and a ticket costs US$190 (as of August 2019), which is relatively expensive for villagers. Another method for reaching the island is by sea travel. Although the shipping fee is much cheaper than airfare at approximately US$12, the route accommodates very few ships. This geographical isolation may have attracted researchers, and various types of research have been conducted on the island [9,10,11,12,13]. Conversely, the geographical isolation of the island is now a challenge for public health. In particular, FSM has serious health concerns about obesity and non-communicable diseases (NCDs) due to the shift in the diet, that is, from a traditional diet mainly based on local crops, marine products, fruits, and sugarcane to the modernized diet, which is heavily dependent on imported food, such as rice, ramen, canned goods, turkey tail, sweetened juice, and snacks [14,15]. According to the WHO, NCDs are estimated to account for 75.0% of all deaths in 2016 [16]. However, little data related to health in Pingelap has been reported because people living in the outer island have been excluded as targets of large-scale population-based surveys. In addition, delivering health care services to the outer islands is extremely difficult due to the limited access [17]; medical resources from the outer islands are very limited. Taking Pingelap as an example, only one small clinic is available, and the island lacks hospitals or medical doctors, so early prevention rather than treatment is required.

Therefore, the study aims to elucidate the current health situation of the people living in Pingelap and investigate the impact of geographical isolation on health, such as for overweight/obesity and high blood pressure. For this reason, the health situations between the Pingelap and Mand communities are compared because their genetic background may be similar. In addition, the study aims to examine the impact of diet, which is considered as the cause of the abovementioned health issues in the country. The study hypothesizes that the people living in Pingelap consume less imported food and continue to rely more on local crops and marine products than the people living in Mand. Thus, their health situation may be relatively better. The study seeks to bring insight into the impact of living in geographically isolated areas on health and diet and clarify the health situation of people living in places where medical resources are relatively poor for early prevention and treatment of NCDs, which is commonly witnessed around the world.

## 2. Materials and Methods

### 2.1. Study Site and Data Collection

**Figure 1:**
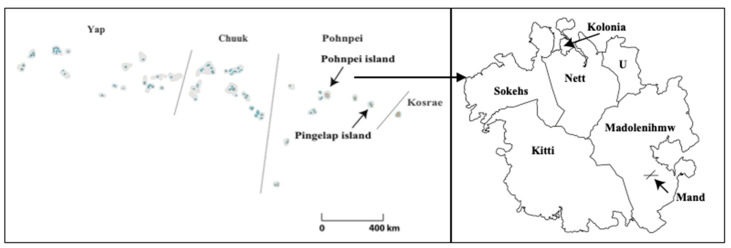
Geographic location of Pingelap Island and Mand.

The study sites were Pingelap and Mand. To reiterate, Pingelap is one of the outer islands of Pohnpei state, whereas Mand is located in a rural municipality in Pohnpei (Figure 1). Several differences in the living environment between Mand and Pingelap were observed in terms of location and distance from the sea. According to the latest population census in 2010, the numbers of households and population were 81 and 459 in Mand and 71 and 258 in Pingelap, respectively [18]. The Pingelapese population has been decreasing in Pingelap and Pohnpei, whereas a large number of Pingelapese ventured abroad and are now living in Guam, Hawaii, and the U.S. mainland.

Data collection was conducted from August to September 2019. Research assistants who fluently speak English and Pingelapese carried out face-to-face interviews with questionnaires. A total of 50 individuals (males and females) aged over 18 years joined from each study site. The sample sizes were small due to logistics. Initially, 50 households were randomly selected from each site based on the lists of residents given by the Department of Health Services of Pohnpei state (Pingelap) and a health assistant (Mand). Then, one family member from each household was selected via the Kish method [19]. Two participants in Mand were excluded due to a different ethnic background (i.e., not from Pingelap).

### 2.2. Anthropometric Measurement

Height was measured without shoes to the first decimal in centimeters using a portable stadiometer (SECA213, SECA, Japan). Weight was measured without shoes using a digital scale (BC-751, Tanita, Japan) with a precision of 0.1 and 0.2 kg for less than 100 and 100–150 kg, respectively. BMI was computed as weight (in kg)/squared height (in m). BMI is widely used to classify overweight and obesity in adults. For adults, BMIs equal to or over 25 and 30 kg/m^2^ were categorized as overweight and obese, respectively [20]. A digital sphygmomanometer (HEM-7210, Omron, Japan) was used to measure systolic blood pressure (SBP) and diastolic blood pressure (DBP). Sitting blood pressure was measured with an arm cuff twice or thrice within 1 h at the longest. The average of the measurement results was used for analysis. According to the WHO, high blood pressure was recorded when an individual’s SBP was equal to or over 140 mm Hg and/or DBP is equal to or over 90 mm Hg on two different days [21].

### 2.3. Dietary Assessment

The aim of the dietary surveys was to examine nutritional intake and habitual diet. For each assessment, the 24-h dietary recall and food frequency questionnaire (FFQ) were applied. The 24-h dietary recall is the standard method for assessing respondents in terms of what and how much food was consumed in the previous 24 h. In the present study, the 24-h dietary recall was conducted within one day using the Automated Multiple-Pass Method [22]. Total energy intake (MJ), protein intake (g), fat intake (g), and carbohydrate intake (g) were calculated using The Pacific Islands Food Composition Tables Second Edition and USDA Food Composition Database after collecting data [23,24]. The proportions of energy intake from proteins, fat, and carbohydrates to total energy intake were also calculated. FFQ is frequently used to evaluate the frequency of food and beverage consumption over a certain period [22]. A preliminary study was conducted in Pohnpei to develop the FFQ, which was applied in the present study. Food items frequently reported by the participants of the FFQ were extracted and assigned different scales [25]. Details of this survey, however, are yet to be published elsewhere. In the present study, the frequencies of consumption of 19 food and beverage items were rated using a 7-point Likert-type scale ranging from 1 = every day (more than three times a day) to 7 = never, and those of the three groups of food were rated using a 4-point Likert-type scale ranging from 1 = frequently to 4 = never.

### 2.4. Data Analysis

Analyses were performed with R version 3.6.3 [26]. Significance level was set at 0.05. Analysis was separately conducted by gender because it is well-known to influence food intake and health status [27,28]. Welch’s *t*-test was used to compare the quantitative measures between regions. To match the direction of the FFQ results with other data, scores of FFQ results were given reverse order, as shown in Table 1. The likelihood ratio test was used to evaluate the difference between regions, in which the null hypothesis was that the ordered logistic regression model to explain the scores by region had the same likelihood as the model to explain the scores by constant.

### 2.5. Ethical Approval

The present study was conducted according to the guidelines of the Declaration of Helsinki, and all procedures involving the participants were approved by the ethical committee of the Graduate School of International Cooperation Studies in Kobe University and the Department of Health and Social Affairs in Pohnpei. Written informed consent was obtained from all participants.

## 3. Results

### 3.1. Demographic Information and Behavior Data about the Sample

Table 2 shows the demographic information and behavior data of subjects by community. A total of 98 participants were enrolled in the study. The mean age of all participants was 42.0 ± 15.4 years. The most common employment category was unemployed in Mand and government employees in Pingelap males and non-government employees for Pingelap females. However, in terms of household income, the Pingelapese participants tended to belong to the “less than 25 US$/2 weeks” category. Approximately half of the participants received remittance from family members working abroad. The mean remittance amount of Pingelap was lower than that in Mand. Similarly, the food expense (amount paid for food per two weeks per household) was relatively lower in Pingelap than Mand. The prevalence of a smoking habit and alcohol consumption were also low in Pingelap for males and females. However, the prevalence of a smoking habit for females was much smaller than males in both regions.

### 3.2. Health Outcome

Table 3 provides the result of the anthropometric measurement. All comparisons showed no statistical significance. However, it was possible to find some tendencies as follows. The mean BMI was higher than 25 kg/m^2^, which was regarded as overweight for every region and for both genders. The mean BMI was significantly higher in females than in males (*p* < 0.001). Among males, the BMI was similar between Mand and Pingelap. Among females, a relatively higher average BMI was observed in Pingelap than in Mand. Of the males, 36.8% in Mand and 26.6% in Pingelap were classified as obese (BMI ≥ 30 kg/m^2^), whereas 75.9% and 85.0% of the females in Mand and Pingelap, respectively, were classified as obese. In terms of blood pressure, contrary to the BMI results, males displayed higher SBP and DBP than females in both Mand and Pingelap with a significant difference only in SBP (*p* = 0.01). Among the males, SBP was higher in Pingelap than in Mand. However, among females, SBP was lower in Pingelap than in Mand. The result for DBP was similar regardless of region. Overall, 26.3% and 17.2% in Mand, and 43.3% and 10.0% in Pingelap, for males and for females, respectively, were classified as SBP ≥ 140 mm Hg and/or DBP ≥ 90 mm Hg.

### 3.3. Diet

Table 4 summarizes the estimated nutritional intake. Total energy intake tended to be lower in Pingelap than in Mand for both genders. The proportion of protein and fat also tended to be lower in Pingelap than Mand, whereas that of carbohydrate was higher in Pingelap than in Mand for both genders. However, no significant regional difference was observed in energy intake and macronutrient intake regardless of gender with one exception. Only the proportion of protein contribution to energy intake among males was significantly lower in Pingelap than in Mand.

Table 5 presents the FFQ result: the higher the score, the higher the intake frequency. Regarding local food items, local crops (i.e., yam, taro, and breadfruit) and banana (including fried banana) were consumed less frequently in Pingelap than in Mand, although a significant regional difference was not found. Coconut juice was more frequently consumed in Pingelap. However, vegetables were rarely eaten in Pingelap, and the consumption frequency of vegetables was significantly lower in Pingelap than in Mand for both males and females.

In the same light as the local food items, no significant difference was observed in the consumption of several imported food items, such as rice, ramen and noodles, bread, and doughnuts. Rice was consumed more frequently than the local crops for both regions. The consumption frequencies of potato chips/snacks and sweetened juice differed across regions, and such items were rarely consumed in Pingelap. Canned fish was also significantly less commonly eaten in Pingelap, whereas the consumption of canned meat did not display a regional difference.

With regard to fish and meat items, sashimi was the most commonly consumed of the other fish items (i.e., sashimi, fried fish, and fish in soup) in Mand. Alternatively, fried fish was the most common way of eating fish in Pingelap, which was consumed significantly more in Pingelap than Mand. Fried pork was rarely eaten in either Mand or Pingelap without a significant difference. In contrast to fried pork, the consumption of fried chicken indicated a significant difference, which was consumed less in Pingelap than in Mand.

Regarding alcohol and sakau, the consumption of alcohol was significantly higher in Mand than in Pingelap for both genders. Sakau is a beverage made from *Piper methysticum* and widely consumed in Oceania. It does not contain alcohol but does have a sedative effect. In FSM, only Pohnpei has a custom of drinking sakau. Thus, the drink was consumed less in Pingelap.

## 4. Discussion

The study investigated the health situation of people living in Pingelap and the impact of living in the geographically isolated area on the health and diet. The study setting was ideal for testing such an impact because the participants had the same ethnic background. The findings suggested that residents in the two sites showed health risks, such as obesity and high blood pressure, which lead to NCDs. Especially, females had higher BMIs, whereas males tended to have high blood pressure. However, contrary to the study hypothesis, the prevalence of obesity in females and high blood pressure in males was higher in Pingelap than in Mand. In addition, the result did not conform to our expectations regarding diet because no significant regional differences were noted in energy intake and consumption of local crops and several imported food items; geographical location influenced food availability in both study sites.

### 4.1. Health Outcome

To the best of our knowledge, the study is the first to report the health situation of the population living in the outer islands of Pohnpei state. The result indicates no regional difference in BMI contrary to the hypothesis. Several previous studies with settings that are similar to those of the current study were conducted in other areas of the Pacific. For example, Hodge et al. (1997) examined BMI in the Polynesian population of Western Samoa across three regions, namely, Apia (urban area), Poutasi (peri-urban area), and Tuasivi (isolated area). The result suggested that the mean BMI was significantly lower in Tuasivi than Poutasi and Apia for males (28.2 kg/m^2^ vs. 29.2 kg/m^2^ vs. 30.4 kg/m^2^) and females (30.0 kg/m^2^ vs. 31.4 kg/m^2^ vs. 33.2 kg/m^2^) [29]. Moreover, Olszowy et al. (2015) investigated adult body composition in a Melanesian population on four islands in Vanuatu, namely, Efate (urban area), Nguna (rural area with urban access), Aneityum (rural area with tourism), and Ambae (rural area). The authors also found that the mean BMI was significantly lower in the most rural area, Ambae (23.4 and 23.1 kg/m^2^), compared with Aneityum (24.6 and 25. 3 kg/m^2^), Nguna (24.6 and 26.3 kg/m^2^), and Efate (26.5 and 29.3 kg/m) for males and females, respectively [30]. The cited studies did not state the impact of living in isolated areas; however, populations with limited access to urban areas displayed lower BMIs. This tendency was also found in a recent study of other Melanesian populations [31,32,33], which was contrary to the findings of the present study. Furthermore, the current study analyzed the data of the WHO Steps survey in 2008, where 2227 residents (males = 900; females = 1327) of Pohnpei island participated to compare our results. After excluding the data outliers, the mean BMIs were 26.6 and 29.5 kg/m^2^ for males and females, respectively [34]. Kobayashi surveyed 118 male and 140 female teachers at public elementary schools in Pohnpei island in 2014 using a self-reported questionnaire. The author reported mean BMI values of 31.7 and 32.5 kg/m^2^ for males and females, respectively [35]. Compared with previous studies, the result of the present study pointed to similar mean BMI levels for males and higher BMI levels for females. Given that all participants had the same ethnic background, genetic background may be one of the factors for the high prevalence of obesity of females in the study population. According to Neel’s thrifty genotype hypothesis, populations that experienced repeated starvation during long voyages and natural disasters are able to utilize food efficiently, thereby causing rapid weight gain when food is steadily supplied in sufficient amounts. This tendency can lead to increased risk of obesity [36]. Although an argument has been presented regarding the hypothesis [37], Neel’s thrifty genotype hypothesis was supported with regard to the Pacific population by the population–genetic study of Furusawa et al. (2010). The study proved that the non-synonymous single nucleotide polymorphisms of the leptin receptor gene Q223R are a genetic factor responsible for the increased prevalence of obesity [38]. As mentioned in the introduction, Pingelap historically experienced severe typhoons several times and famine following these disasters, such that people may have thrifty genes. In addition, Pingelap underwent a population bottleneck; thus, a possibility exists that people who survived the bottleneck have a high frequency of thrifty genes and are likely to gain weight.

The results indicated no regional difference in blood pressure. The prevalence of hypertension among Pingelap males was highest in the study population, which was contrary to the hypothesis. In addition, the results are not in accordance with those of previous studies. Taylor et al. (1987) compared high blood pressure in Polynesian populations across two sites: Noumea (New Caledonia), which was highly urbanized and fully integrated into the modernized society and cash economy, and Wallis Island, where most of the population engaged in subsistence agriculture and fishing and import of certain food. The findings revealed that the mean SBPs and DBPs were significantly lower in Wallis (117.4/72.9 and 116.3/73.4 mm Hg) compared with Noumea (123.6/77.6 and 123.9/79.1 mm Hg) for males and females, respectively [39]. This tendency was demonstrated in the Melanesian population [32]. The mean SBPs and DBPs of the WHO Steps survey in 2008 were 129.3/76.5 and 123.1/74.4 mm Hg for males and females, respectively. However, the results were lower compared with the findings of the present study. In addition, the prevalence of hypertension (SBP ≥ 140 mm Hg and/or DBP ≥ 90 mm Hg) as cited by the survey was 24.6% and 20.0% for males and females, respectively [34], which was higher than the result of the present study for males, especially in Pingelap (43.3%). In general, males are at greater risk for high blood pressure than females, which is consistent with the current result [40,41]. In addition, smoking and alcohol consumption are risk factors for elevated blood pressure and NCDs [42,43]. The prevalence of smoking and alcohol consumption experience was relatively higher for males (Table 2) regardless of region. However, that of Mand was higher than that of Pingelap. In addition, the FFQ result indicates that the consumption frequency of alcohol was low in Pingelap for both genders. The result illustrated that 42.1% and 13% of males and females, respectively, drunk alcohol at least once a week in Mand, whereas only 10% of males in Pingelap drunk at least once a week. This result may strongly relate to a social rule and cultural norm, that is, people are prohibited from drinking alcohol on Pingelap island due to their Christianity. Although, in fact, males secretly made alcohol drinks from yeast, water, and sugar available from kiosks and enjoyed drinking, the frequency was low. High salt intake is also a well-known factor of high blood pressure [44]. However, the present study overlooked the salt intake of the population. Thus, this aspect can serve as another avenue for future studies.

### 4.2. Diet

#### 4.2.1. Geographical Condition and Food Availability

The findings suggested that geographical location influenced food availability. The present study proposed that the proportion of protein contribution to energy intake among males was lower in Pingelap, which may be influenced by the availability of protein-rich food. Protein sources are typically limited to marine products in Pingelap. Although people raise pigs, pork is eaten on special occasions, such as feasts, wedding parties, and funerals. In addition, only two small kiosks on the island sell canned products. Meanwhile, residents in Mand can easily purchase marine and meat products, including frozen and canned products from local markets and supermarkets in town or kiosks in the community. The low availability of meat products in Pingelap may be related to the lower consumption of fried chicken. Pork is also reserved for special occasions in Mand, which may result in the low consumption of fried pork for both regions. The consumption of canned fish was higher in Mand, which may be because the people in Mand had no direct access to the sea. In contrast, Pingelap was surrounded by sea, and people fish and share their fare with the residents. This difference also reflected the difference in the price of raw fish, which was US$0.5/lb in Pingelap and US$1.75/lb in Mand in August 2019. In addition to fresh fish, residents in Pingelap regularly hunt for land crab or coconut crab in the jungles. The various availabilities of these items may be related to the lower consumption of canned fish. However, no significant difference was observed in the consumption of sashimi. The reason for this result may be because they are sold in the stall along roads in Pohnpei for approximately US$1–2, and people in Mand easily purchase them. Conversely, fried fish was more commonly consumed in Pingelap. A villager in Pingelap stated that the majority of the villagers did not own refrigerators on the island, so they tend to fry fish for longer storage. In fact, a previous study revealed that less than 10% of villagers owned refrigerators in Pingelap [45]. This fact may be related to the finding. Contrary to the hypothesis, the participants in Pingelap consumed canned meat as frequently as those in Mand. Kawai et al. (2010) compared the diet (frequency of eating each food item) between Pohnpei, Pingelap, and Mokil, which is one of the other outer islands in Pohnpei, in 2008, and found that canned meat was significantly less consumed in Pingelap than Pohnpei [45]. This result suggests that the consumption of canned meat in Pingelap has increased. However, Sacks (1988) wrote that Spam was served every meal-time when he visited Pingelap in 1994. Therefore, Spam may have been consumed in the past [5]. In addition, the availability of other food items may be associated with the consumption of other food items, such as potato chips or snacks, sweetened juices, vegetables, and sakau. Similar to meat products, residents in Mand easily purchase potato chips or snacks, sweetened juices, and vegetables, whereas these food items are rarely sold in Pingelap. The questionnaire posed questions regarding the food items that the participants eat as snacks. The majority of the participants in Mand answered “chocolate”, “candy”, and “potato chips”, whereas those in Pingelap answered “banana”, “pandanus”, and “papaya”. These answers support the current result. The lower consumption of vegetables in Pingelap may be also due to the lack of custom of eating vegetables in addition to availability. Yamamoto et al. [10] conducted a dietary survey for 18 months in Pingelap and found that people rarely ate vegetables, especially green or leafy ones. A 60-year old woman who works as a specialist in nutrition in the College of Micronesia relayed that people in FSM are unused to eating vegetables because they were originally food for livestock. The case is true for people living not only in Pingelap but also in FSM. Sakau was also unavailable in Pingelap because the environment of the atoll is unsuitable for growing *P. methysticum* [46].

#### 4.2.2. Modernized Diet

Contrary to the hypothesis, no significant difference was observed in the consumption of local crops and several imported food items. Evidently, rice is the main staple regardless of region, which is consistent with previous studies in Pohnpei [47,48]. Sacks (1998) stated that the diet in Pingelap is dependent on local crops and tuna [5]. However, today, local crops have been replaced by rice, and only when rice is unavailable did people eat local crops. A health assistant who is working in the dispensary in Pingelap stated that a couple of decades ago, people in Pingelap heavily relied on local crops, especially taro. Only when taro was unavailable did people eat rice. In addition, the study population in Pingelap consumed other imported food items, such as ramen and noodles, bread, and doughnuts, as commonly as those in Mand. Eating bread and doughnuts entails the purchase of yeast, flour, and baking powder from kiosks, and residents have to bake bread themselves in Pingelap. In contrast, those in Mand purchase bread and doughnuts from kiosks and in town. This result suggests that the diet of Pingelap has changed; thus, they can afford to buy imported food items. Remittance from family members may play a significant role in such a change. Another possibility is that many household chores have been reduced, which enabled people to allot time for baking bread and doughnuts.

The data suggested that the consumption of imported foods has increased in Pingelap. In other words, the diet has gradually been modernized. The study is cross-sectional in nature, so the causal relationship between diet and health remains unclear. However, a previous study suggested that the change toward a western diet could markedly contribute to the increased prevalence of obesity [31]. In addition, the thrifty gene is considered to function efficiently in combination with environmental factors, such as changes in diet and physical activity [49,50,51]. Especially, females are more at risk for fat deposition in the modern environment, where high-calorie, high-fat food is becoming available due to modernization and an increased number of imported food items [30,52,53]. Therefore, the change in the diet of the study population could be the trigger that increased the prevalence of overweight and obesity with a combination of the thrifty gene. Moreover, several imported food items, particularly processed foods and fast food, contain excessive salt, which may be related to elevated blood pressure [54]. Further studies are required to investigate the impact of genetics and diet on the health situation of the study population.

### 4.3. Limitation

First, a single-day dataset from the 24-h dietary recall may not represent the usual intake in general [55]. In addition, several studies revealed that people tend to underestimate food and portion size, especially for foods that are considered less healthy [56]. Many factors were revealed to be associated with performance on recall. Lichtman et al. suggested that obese individuals are more likely to underreport food intake and portion size [57]. Thus, a possibility exists that nutrition intake based on the 24-h dietary recall may be underestimated. However, single-day data may be used to examine nutrition intake by group level. Second, diet in Pingelap may be heavily influenced by the survey period. Yamamoto et al. cited that no rice stocks were observed on the island during certain periods in their survey [10]. Moreover, FFQ was developed through a preliminary survey conducted in Pohnpei. Therefore, this result may not completely reflect the diet of Pingelap. Third, the sample size was relatively small, such that the sample may not be fully representative of all the residents in the study sites. Lastly, hypertension is frequently diagnosed based on measured values for two days. However, the measurements were repeated within the same day in the present study. Therefore, the possibility exists that blood pressure was overestimated due to the mental stress of suffering from extraordinal experiences.

## 5. Conclusions

The present study did not find the impact of living in geographically isolated areas on health and diet. The results suggested the majority of the Pingelapese had a higher risk factor burden for NCDs, such as for overweight/obesity and high blood pressure. Females, in particular, had a high prevalence of obesity. Regarding diet, the findings demonstrated that geographic location influenced food availability. However, imported foods were commonly consumed even in Pingelap which has limited access to the food market, although they continued to depend on subsistence fishing regarding marine products. The results have important implications for research on and intervention strategies for public health, as a high prevalence of NCD risk factors was found for a place with limited public health resources. To understand the causes of such health issues in Pingelap, further studies are required from multiple perspectives, such as genetics and physiology. In addition, future results may be beneficial as clues for the early implementation of prevention strategies, which may help people avoid the abovementioned health burdens.

## Figures and Tables

**Table 1:** Food items and scores used at interview and statistical analysis of food frequency questionnaire (FFQ).

Food Item	Scores in Statistical Analysis	
Rice, local crops, coffee/tea	1	Never
	2	Seldom
	3	Less than once a day, but sometimes in a week
	4	Once a day
	5	Twice a day
	6	3 times a day
	7	More than 3 times a day
Ramen and noodles, bananas, bread, doughnuts and sweetened bread, potato chips/snacks, sashimi, fried fish, fish in soup, canned fish, fried chicken, fried pork, canned meat, coconut juice, coke, soda/other sweetened juice alcohol drink, sakau	1	Never
	2	Seldom
	3	Less than once a week, but sometimes in a month
	4	1–2 times a week
	5	3–4 times a week
	6	5–6 times a week
	7	Every day
Fried banana, meal cooked with coconut milk, vegetables	1	Never
	2	Seldom
	3	Sometimes
	4	Frequently

**Table 2:** Data on demographic information and behavior of the participants from the two communities according to gender.

		Male				Female			
		Mand (N = 19)		Pingelap (N = 30)		Mand (N = 29)		Pingelap (N = 20)	
Age	Mean ± SD	41.3 ± 14.4		45.0 ± 15.7		37.7 ± 15.9		44.2 ± 14.7	
Ethnicity	Pingelapese	19	(100.0)	30	(100.0)	29	(100.0)	20	(100.0)
Marital status	Married	10	(52.6)	16	(53.3)	24	(82.8)	17	(85.0)
	Single, never married	8	(42.1)	9	(30.0)	4	(13.8)	3	(15.0)
	Widowed	1	(5.3)	5	(16.7)	1	(3.4)	0	(0.0)
Family member	1~2	10	(52.6)	12	(40.0)	9	(31.0)	3	(15.0)
	3~5	9	(47.4)	12	(40.0)	17	(58.6)	15	(75.0)
	More than 6	0	(0.0)	6	(20.0)	3	(10.3)	2	(10.0)
Education achievement	Elementary school graduated	8	(42.1)	12	(40.0)	8	(27.6)	8	(40.0)
	High school graduated	2	(10.5)	14	(46.7)	13	(44.8)	10	(50.0)
	2/4-year-college graduated	4	(21.1)	4	(13.3)	8	(27.6)	2	(10.0)
	Professional (graduate, postgraduate)	5	(26.3)	0	(0.0)	0	(0.0)	0	(0.0)
Employment status	Self-employed	4	(21.1)	3	(10.0)	7	(24.1)	0	(0.0)
	Government employee	6	(31.6)	9	(30.0)	1	(3.4)	6	(30.0)
	Non-government employee	0	(0.0)	3	(10.0)	2	(6.9)	8	(40.0)
	Housemaker	0	(0.0)	3	(10.0)	4	(13.8)	0	(0.0)
	Retired	1	(5.3)	4	(13.3)	0	(0.0)	2	(10.0)
	Unemployed	8	(42.1)	8	(26.7)	15	(51.7)	4	(20.0)
Household income (US$ per 2 weeks)	Less than 25	5	(26.3)	10	(33.3)	3	(10.3)	3	(15.0)
	25–100	2	(10.5)	10	(33.3)	4	(13.8)	6	(30.0)
	100–250	7	(36.8)	4	(13.3)	12	(41.4)	2	(10.0)
	250–500	4	(21.1)	5	(16.7)	5	(17.2)	7	(35.0)
	500–750	1	(5.3)	0	(0.0)	0	(0.0)	0	(0.0)
	More than 750	0	(0.0)	1	(3.3)	3	(10.3)	0	(0.0)
	Do not know	0	(0.0)	0	(0.0)	2	(6.9)	2	(10.0)
Remittance	Yes (regularly)	8	(42.1)	18	(60.0)	24	(82.8)	9	(45.0)
	No	10	(52.6)	12	(40.0)	2	(6.9)	7	(35.0)
	Sometimes	1	(5.3)	0	(0.0)	3	(10.3)	4	(20.0)
Remittance amount (US$ per month)	Mean ± SD	271.9 ± 116.1		204.4 ± 157.2		392.1 ± 249.0		205.6 ± 115.8	
Food expense (US$ per 2 weeks)	Mean ± SD	107.6 ± 74.2		51.9 ± 51.5		129.3 ± 93.0		61.0 ± 32.1	
Smoking habit	Yes	14	(73.7)	19	(63.3)	6	(20.7)	0	(0.0)
	No	5	(26.3)	11	(36.7)	23	(79.3)	20	(100.0)
Experience of alcohol consumption (have drunk any alcohol in lifetime)	Yes	15	(78.9)	25	(83.3)	13	(44.8)	3	(15.0)
	No	3	(15.8)	5	(16.7)	16	(55.2)	17	(85.0)
	Underage	1	(5.3)	0	(0.0)	0	(0.0)	0	(0.0)

**Table 3:** The comparison of anthropometric measurements between two communities separately for males and females (mean ± SD).

	Male							Female						
	Mand (N = 19)			Pingelap (N = 30)			*p*-Value	Mand (N = 29)			Pingelap (N = 20)			*p*-Value
Height (cm)	160.7	±	5.0	163.4	±	6.5	0.11	151.2	±	5.4	150.5	±	5.3	0.64
Weight (kg)	71.4	±	16.1	73.4	±	13.6	0.65	80.8	±	19.7	82.5	±	15.1	0.74
BMI	27.6	±	6.3	27.4	±	4.3	0.88	35.2	±	7.4	36.5	±	6.4	0.52
SBP (mmHg)	131.8	±	17.8	136.5	±	13.8	0.34	128.8	±	14.5	123.4	±	8.0	0.23
DBP (mmHg)	76.0	±	11.4	75.6	±	6.8	0.91	76.4	±	9.8	75.3	±	8.0	0.69

Note: *p*-value is the result of Welch’s *t*-test. Number of participants of females in Pingelap for height, weight, and BMI was 19 because one participant was not able to measure. Number of participants of females in Mand for blood pressure was 27 because two participants were not able to measure due to the size of arm cuff. SBP—systolic blood pressure (SBP), DBP—diastolic blood pressure.

**Table 4:** Comparison of the estimated nutrition intake from 24-h dietary recall between two communities separately for males and females (mean ± SD).

	Male							Female						
	Mand (N = 19)			Pingelap (N = 30)			*p*-Value	Mand (N = 29)			Pingelap (N = 20)			*p*-Value
Energy intake (MJ)	8.6	±	3.6	7.9	±	2.9	0.46	8.0	±	2.6	7.7	±	2.7	0.76
Protein intake (g)	85.5	±	49.6	62.3	±	28.5	0.07	74.6	±	24.2	63.8	±	25.5	0.14
Fat intake (g)	58.9	±	44.2	48.8	±	42.7	0.29	59.0	±	25.8	48.6	±	29.4	0.21
Carbohydrate intake (g)	295.5	±	95.0	296.6	±	89.6	0.97	266.8	±	111.0	285.6	±	124.8	0.59
Proportion of protein (%)	16.3	±	4.2	13.5	±	4.7	0.03	16.2	±	3.9	14.0	±	4.5	0.09
Proportion of fat (%)	24.0	±	8.7	20.9	±	10.2	0.27	28.2	±	10.4	23.6	±	12.6	0.18
Proportion of carbohydrate (%)	59.5	±	11.3	65.2	±	9.8	0.08	55.3	±	12.5	61.8	±	13.6	0.10

Note: *p*-value was calculated by Welch’s *t*-test.

**Table 5:** Comparison of habitual diet from food frequency questionnaire between two communities separately for men and women (median (interquartile range)).

	Male			Female		
	Mand (N = 19)	Pingelap (N = 30)	*p*-Value	Mand (N = 29)	Pingelap (N = 20)	*p*-Value
Rice	6.0 (5.0–6.0)	5.0 (5.0–6.0)	0.61	6.0 (5.0–6.0)	6.0 (5.0–6.0)	1.0
Local crops	4.0 (4.0–5.0)	4.0 (2.0–5.0)	0.25	4.0 (3.0–5.0)	3.5 (3.0–5.0)	0.67
Coffee/Tea	4.0 (2.0–5.0)	5.0 (4.0–6.0)	0.07	4.0 (1.0–4.0)	4.0 (2.0–4.0)	0.55
Ramen & Noodle	4.0 (4.0–5.0)	4.0 (4.0–5.0)	0.28	4.0 (4.0–5.0)	4.0 (3.8–5.0)	0.24
Banana	4.0 (4.0–5.0)	5.0 (4.0–5.0)	0.64	4.0 (4.0–5.0)	4.0 (4.0–5.0)	0.11
Bread	4.0 (4.0–6.0)	4.5 (4.0–5.0)	0.46	5.0 (4.0–6.0)	4.5 (4.0–5.0)	0.14
Doughnut & Sweetened bread	4.0 (3.5–5.5)	4.0 (3.3–5.0)	0.27	4.0 (3.0–5.0)	5.0 (3.5–5.0)	0.86
Potato chips/snack	4.0 (3.5–5.0)	1.0 (1.0–2.8)	<0.001	4.0 (4.0–5.0)	2.0 (1.0–4.0)	0.003
Sashimi	5.0 (4.0–6.0)	5.0 (2.5–5.0)	0.25	5.0 (4.0–5.0)	5.0 (2.0–5.0)	0.26
Fried fish	4.0 (4.0–5.0)	5.0 (5.0–6.0)	0.07	4.0 (4.0–5.0)	5.0 (5.0–6.3)	<0.001
Fish in soup	4.0 (2.0–4.5)	4.5 (3.0–5.0)	0.44	4.0 (4.0–5.0)	5.0 (3.0–5.0)	0.68
Canned fish	5.0 (4.0–5.0)	4.0 (2.0–4.0)	0.002	5.0 (4.0–5.0)	4.0 (1.0–4.0)	0.004
Fried chicken	4.0 (4.0–5.0)	2.5 (1.3–4.0)	<0.001	4.0 (4.0–5.0)	2.0 (1.0–4.3)	0.01
Fried pork	2.0 (1.0–3.0)	2.0 (1.0–2.0)	0.21	2.0 (1.0–2.0)	2.0 (1.0–2.0)	0.90
Canned meat	4.0 (2.0–5.0)	4.0 (2.0–4.0)	0.44	4.0 (4.0–5.0)	4.0 (2.8–4.3)	0.53
Coconut juice	4.0 (3.5–5.0)	5.0 (5.0–5.0)	0.02	4.0 (3.0–5.0)	5.0 (4.8–6.3)	0.002
Coke, Soda/sweetened juice	5.0 (4.0–5.0)	4.0 (1.3–4.0)	<0.001	4.0 (4.0–5.0)	4.0 (1.0–4.0)	0.10
Alcohol drink	3.0 (2.0–4.0)	1.0 (1.0–2.0)	0.001	1.0 (1.0–2.0)	1.0 (1.0–1.0)	<0.001
Sakau	2.0 (1.0–3.0)	1.0 (1.0–1.0)	0.003	2.0 (1.0–4.0)	1.0 (1.0–1.0)	<0.001
Fried banana	3.0 (2.0–3.0)	3.0 (1.3–3.0)	0.63	3.0 (2.0–3.0)	3.0 (1.0–3.0)	0.83
Meal cooked with coconut milk	3.0 (3.0–3.0)	3.0 (3.0–3.0)	0.16	3.0 (3.0–3.0)	3.0 (3.0–3.0)	0.60
Vegetable	3.0 (3.0–3.0)	3.0 (1.0–3.0)	0.01	3.0 (3.0–3.0)	3.0 (2.0–3.0)	0.03

Note: *p*-value was calculated by likelihood ratio test.
